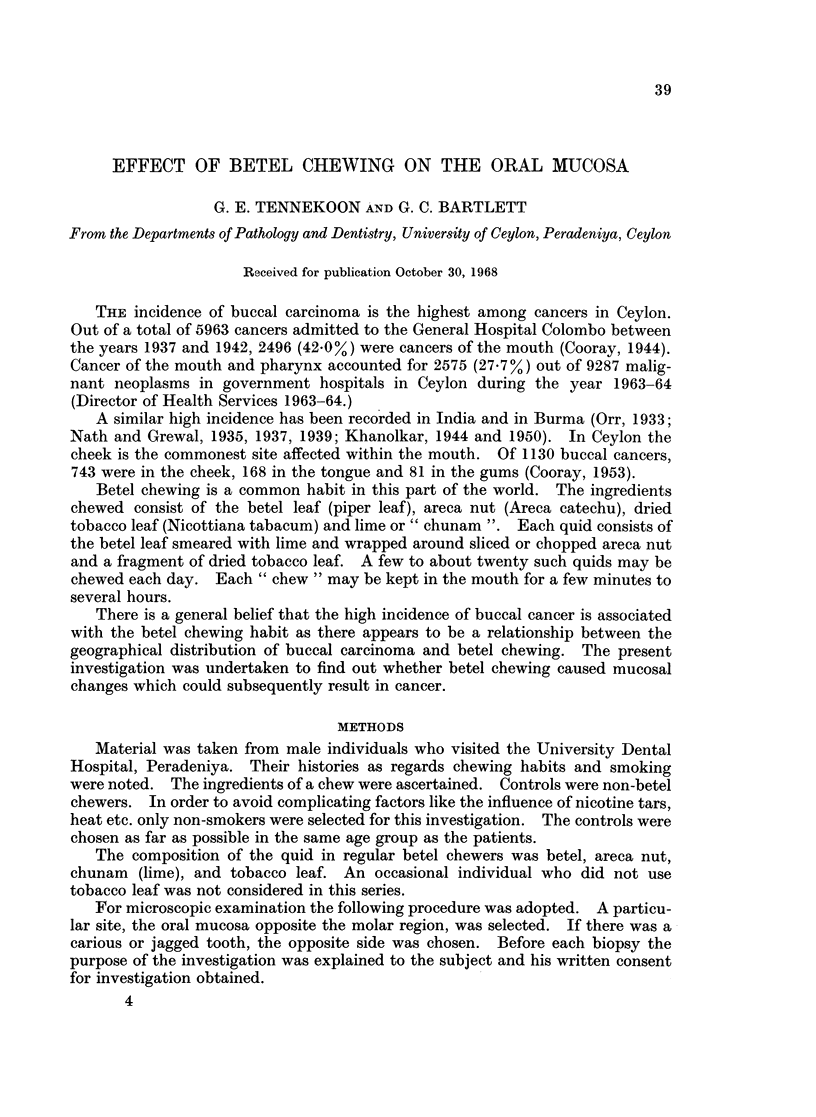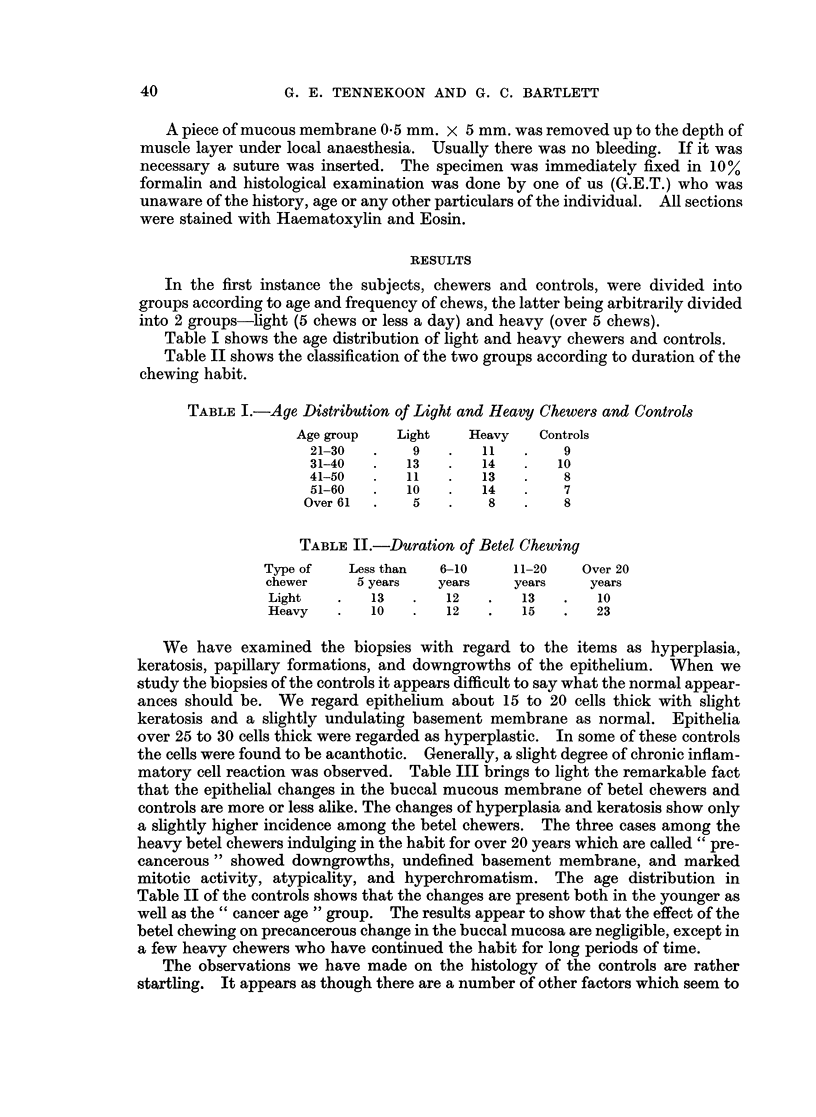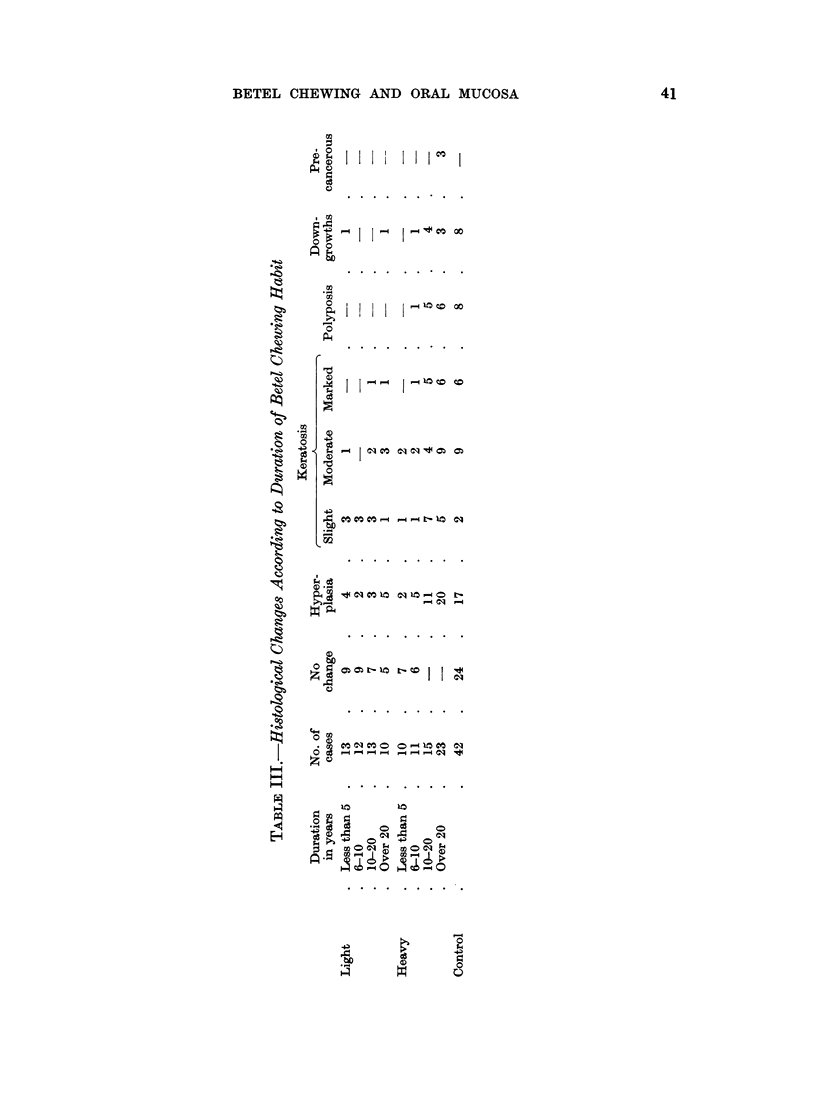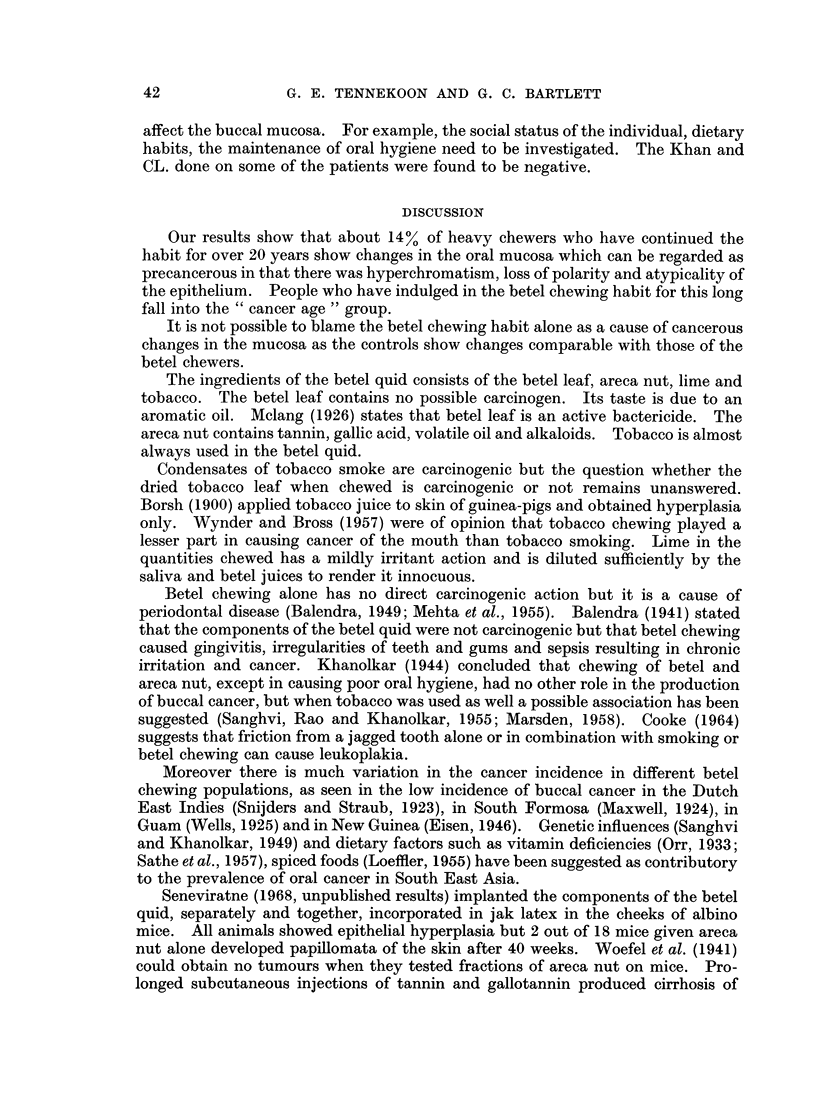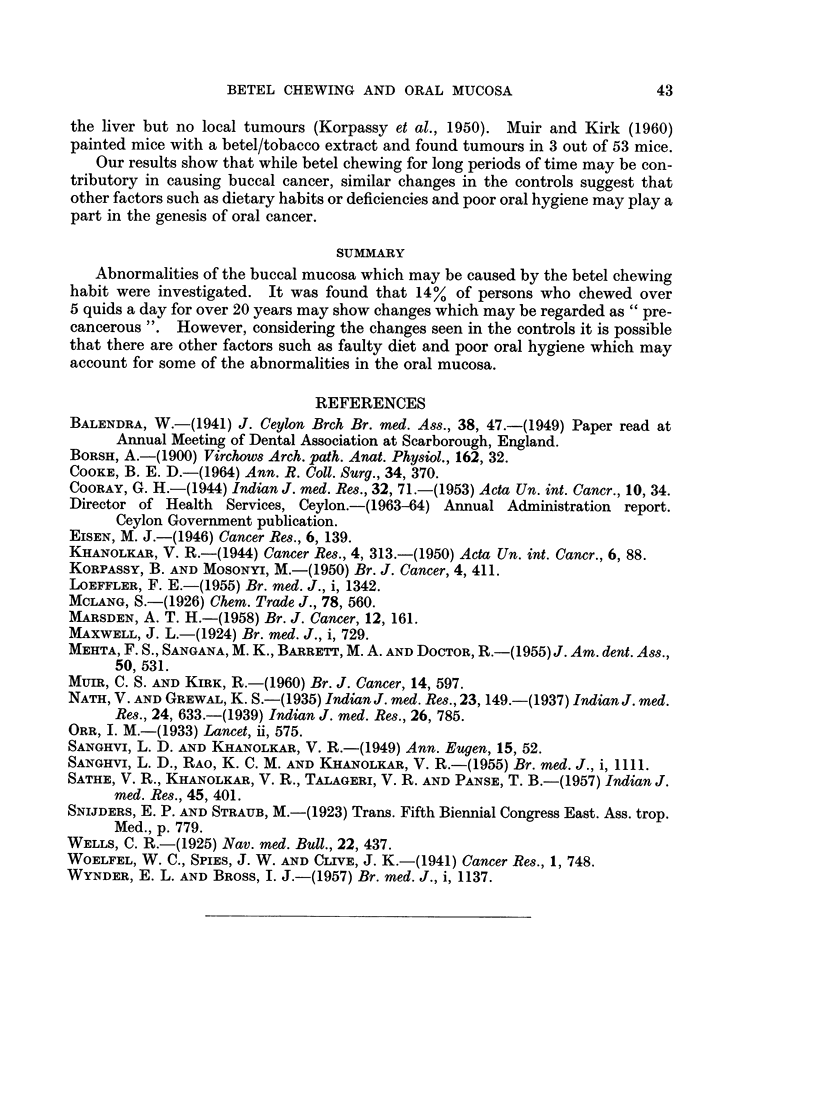# Effect of betel chewing on the oral mucosa.

**DOI:** 10.1038/bjc.1969.6

**Published:** 1969-03

**Authors:** G. E. Tennekoon, G. C. Bartlett


### EFFECT OF BETEL CHEWING ON THE ORAL MUCOSA

G. E. TENNEKOON AND G. C. BARTLETT

*From the Departments of Pathology and Dentistry, University of Ceylon, Peradeniya, Ceylon*

Received for publication October 30, 1968

THE incidence of buccal carcinoma is the highest among cancers in Ceylon. Out of a total of 5963 cancers admitted to the General Hospital Colombo between the years 1937 and 1942, 2496 (42·0%) were cancers of the mouth (Cooray, 1944). Cancer of the mouth and pharynx accounted for 2575 (27·7%) out of 9287 malignant neoplasms in government hospitals in Ceylon during the year 1963–64 (Director of Health Services 1963–64.)

A similar high incidence has been recorded in India and in Burma (Orr, 1933; Nath and Grewal, 1935, 1937, 1939; Khanolkar, 1944 and 1950). In Ceylon the cheek is the commonest site affected within the mouth. Of 1130 buccal cancers, 743 were in the cheek, 168 in the tongue and 81 in the gums (Cooray, 1953).

Betel chewing is a common habit in this part of the world. The ingredients chewed consist of the betel leaf (piper leaf), areca nut (Areca catechu), dried tobacco leaf (Nicottiana tabacum) and lime or " chunam ". Each quid consists of the betel leaf smeared with lime and wrapped around sliced or chopped areca nut and a fragment of dried tobacco leaf. A few to about twenty such quids may be chewed each day. Each " chew " may be kept in the mouth for a few minutes to several hours.

There is a general belief that the high incidence of buccal cancer is associated with the betel chewing habit as there appears to be a relationship between the geographical distribution of buccal carcinoma and betel chewing. The present investigation was undertaken to find out whether betel chewing caused mucosal changes which could subsequently result in cancer.

### METHODS

Material was taken from male individuals who visited the University Dental Hospital, Peradeniya. Their histories as regards chewing habits and smoking were noted. The ingredients of a chew were ascertained. Controls were non-betel chewers. In order to avoid complicating factors like the influence of nicotine tars, heat etc. only non-smokers were selected for this investigation. The controls were chosen as far as possible in the same age group as the patients.

The composition of the quid in regular betel chewers was betel, areca nut, chunam (lime), and tobacco leaf. An occasional individual who did not use tobacco leaf was not considered in this series.

For microscopic examination the following procedure was adopted. A particular site, the oral mucosa opposite the molar region, was selected. If there was a carious or jagged tooth, the opposite side was chosen. Before each biopsy the purpose of the investigation was explained to the subject and his written consent for investigation obtained.

A piece of mucous membrane 0·5 mm. × 5 mm. was removed up to the depth of muscle layer under local anaesthesia. Usually there was no bleeding. If it was necessary a suture was inserted. The specimen was immediately fixed in 10% formalin and histological examination was done by one of us (G.E.T.) who was unaware of the history, age or any other particulars of the individual. All sections were stained with Haematoxylin and Eosin.

### RESULTS

In the first instance the subjects, chewers and controls, were divided into groups according to age and frequency of chews, the latter being arbitrarily divided into 2 groups—light (5 chews or less a day) and heavy (over 5 chews).

Table I shows the age distribution of light and heavy chewers and controls.

Table II shows the classification of the two groups according to duration of the chewing habit.

TABLE I.—*Age Distribution of Light and Heavy Chewers and Controls*

| Age group | Light | Heavy | Controls |
|---|---|---|---|
| 21–30 | 9 | 11 | 9 |
| 31–40 | 13 | 14 | 10 |
| 41–50 | 11 | 13 | 8 |
| 51–60 | 10 | 14 | 7 |
| Over 61 | 5 | 8 | 8 |

TABLE II.—*Duration of Betel Chewing*

| Type of chewer | Less than 5 years | 6–10 years | 11–20 years | Over 20 years |
|---|---|---|---|---|
| Light | 13 | 12 | 13 | 10 |
| Heavy | 10 | 12 | 15 | 23 |

We have examined the biopsies with regard to the items as hyperplasia, keratosis, papillary formations, and downgrowths of the epithelium. When we study the biopsies of the controls it appears difficult to say what the normal appearances should be. We regard epithelium about 15 to 20 cells thick with slight keratosis and a slightly undulating basement membrane as normal. Epithelia over 25 to 30 cells thick were regarded as hyperplastic. In some of these controls the cells were found to be acanthotic. Generally, a slight degree of chronic inflammatory cell reaction was observed. Table III brings to light the remarkable fact that the epithelial changes in the buccal mucous membrane of betel chewers and controls are more or less alike. The changes of hyperplasia and keratosis show only a slightly higher incidence among the betel chewers. The three cases among the heavy betel chewers indulging in the habit for over 20 years which are called " precancerous " showed downgrowths, undefined basement membrane, and marked mitotic activity, atypicality, and hyperchromatism. The age distribution in Table II of the controls shows that the changes are present both in the younger as well as the " cancer age " group. The results appear to show that the effect of the betel chewing on precancerous change in the buccal mucosa are negligible, except in a few heavy chewers who have continued the habit for long periods of time.

The observations we have made on the histology of the controls are rather startling. It appears as though there are a number of other factors which seem to

TABLE III.—*Histological Changes According to Duration of Betel Chewing Habit*

| | Duration in years | No. of cases | No change | Hyper-plasia | Keratosis | | | Polyposis | Down-growths | Pre-cancerous |
|---|---|---|---|---|---|---|---|---|---|---|
| | | | | | Slight | Moderate | Marked | | | |
| Light | Less than 5 | 13 | 9 | 4 | 3 | 1 | — | — | 1 | — |
| | 6–10 | 12 | 9 | 2 | 3 | — | — | — | — | — |
| | 10–20 | 13 | 7 | 3 | 3 | 2 | 1 | — | — | — |
| | Over 20 | 10 | 5 | 5 | 1 | 3 | 1 | — | 1 | — |
| Heavy | Less than 5 | 10 | 7 | 2 | 1 | 2 | — | — | — | — |
| | 6–10 | 11 | 6 | 5 | 1 | 2 | 1 | 1 | 1 | — |
| | 10–20 | 15 | — | 11 | 7 | 4 | 5 | 5 | 4 | — |
| | Over 20 | 23 | — | 20 | 5 | 9 | 6 | 6 | 3 | 3 |
| Control | | 42 | 24 | 17 | 2 | 9 | 6 | 8 | 8 | — |

affect the buccal mucosa. For example, the social status of the individual, dietary habits, the maintenance of oral hygiene need to be investigated. The Khan and CL. done on some of the patients were found to be negative.

### DISCUSSION

Our results show that about 14% of heavy chewers who have continued the habit for over 20 years show changes in the oral mucosa which can be regarded as precancerous in that there was hyperchromatism, loss of polarity and atypicality of the epithelium. People who have indulged in the betel chewing habit for this long fall into the " cancer age " group.

It is not possible to blame the betel chewing habit alone as a cause of cancerous changes in the mucosa as the controls show changes comparable with those of the betel chewers.

The ingredients of the betel quid consists of the betel leaf, areca nut, lime and tobacco. The betel leaf contains no possible carcinogen. Its taste is due to an aromatic oil. Mclang (1926) states that betel leaf is an active bactericide. The areca nut contains tannin, gallic acid, volatile oil and alkaloids. Tobacco is almost always used in the betel quid.

Condensates of tobacco smoke are carcinogenic but the question whether the dried tobacco leaf when chewed is carcinogenic or not remains unanswered. Borsh (1900) applied tobacco juice to skin of guinea-pigs and obtained hyperplasia only. Wynder and Bross (1957) were of opinion that tobacco chewing played a lesser part in causing cancer of the mouth than tobacco smoking. Lime in the quantities chewed has a mildly irritant action and is diluted sufficiently by the saliva and betel juices to render it innocuous.

Betel chewing alone has no direct carcinogenic action but it is a cause of periodontal disease (Balendra, 1949; Mehta *et al.*, 1955). Balendra (1941) stated that the components of the betel quid were not carcinogenic but that betel chewing caused gingivitis, irregularities of teeth and gums and sepsis resulting in chronic irritation and cancer. Khanolkar (1944) concluded that chewing of betel and areca nut, except in causing poor oral hygiene, had no other role in the production of buccal cancer, but when tobacco was used as well a possible association has been suggested (Sanghvi, Rao and Khanolkar, 1955; Marsden, 1958). Cooke (1964) suggests that friction from a jagged tooth alone or in combination with smoking or betel chewing can cause leukoplakia.

Moreover there is much variation in the cancer incidence in different betel chewing populations, as seen in the low incidence of buccal cancer in the Dutch East Indies (Snijders and Straub, 1923), in South Formosa (Maxwell, 1924), in Guam (Wells, 1925) and in New Guinea (Eisen, 1946). Genetic influences (Sanghvi and Khanolkar, 1949) and dietary factors such as vitamin deficiencies (Orr, 1933; Sathe *et al.*, 1957), spiced foods (Loeffler, 1955) have been suggested as contributory to the prevalence of oral cancer in South East Asia.

Seneviratne (1968, unpublished results) implanted the components of the betel quid, separately and together, incorporated in jak latex in the cheeks of albino mice. All animals showed epithelial hyperplasia but 2 out of 18 mice given areca nut alone developed papillomata of the skin after 40 weeks. Woefel *et al.* (1941) could obtain no tumours when they tested fractions of areca nut on mice. Prolonged subcutaneous injections of tannin and gallotannin produced cirrhosis of the liver but no local tumours (Korpassy *et al.*, 1950). Muir and Kirk (1960) painted mice with a betel/tobacco extract and found tumours in 3 out of 53 mice.

Our results show that while betel chewing for long periods of time may be contributory in causing buccal cancer, similar changes in the controls suggest that other factors such as dietary habits or deficiencies and poor oral hygiene may play a part in the genesis of oral cancer.

### SUMMARY

Abnormalities of the buccal mucosa which may be caused by the betel chewing habit were investigated. It was found that 14% of persons who chewed over 5 quids a day for over 20 years may show changes which may be regarded as " precancerous ". However, considering the changes seen in the controls it is possible that there are other factors such as faulty diet and poor oral hygiene which may account for some of the abnormalities in the oral mucosa.

### REFERENCES

BALENDRA, W.—(1941) *J. Ceylon Brch Br. med. Ass.*, **38**, 47.—(1949) Paper read at Annual Meeting of Dental Association at Scarborough, England.

BORSH, A.—(1900) *Virchows Arch. path. Anat. Physiol.*, **162**, 32.

COOKE, B. E. D.—(1964) *Ann. R. Coll. Surg.*, **34**, 370.

COORAY, G. H.—(1944) *Indian J. med. Res.*, **32**, 71.—(1953) *Acta Un. int. Cancr.*, **10**, 34.

Director of Health Services, Ceylon.—(1963–64) Annual Administration report. Ceylon Government publication.

EISEN, M. J.—(1946) *Cancer Res.*, **6**, 139.

KHANOLKAR, V. R.—(1944) *Cancer Res.*, **4**, 313.—(1950) *Acta Un. int. Cancr.*, **6**, 88.

KORPASSY, B. AND MOSONYI, M.—(1950) *Br. J. Cancer*, **4**, 411.

LOEFFLER, F. E.—(1955) *Br. med. J.*, i, 1342.

MCLANG, S.—(1926) *Chem. Trade J.*, **78**, 560.

MARSDEN, A. T. H.—(1958) *Br. J. Cancer*, **12**, 161.

MAXWELL, J. L.—(1924) *Br. med. J.*, i, 729.

MEHTA, F. S., SANGANA, M. K., BARRETT, M. A. AND DOCTOR, R.—(1955) *J. Am. dent. Ass.*, **50**, 531.

MUIR, C. S. AND KIRK, R.—(1960) *Br. J. Cancer*, **14**, 597.

NATH, V. AND GREWAL, K. S.—(1935) *Indian J. med. Res.*, **23**, 149.—(1937) *Indian J. med. Res.*, **24**, 633.—(1939) *Indian J. med. Res.*, **26**, 785.

ORR, I. M.—(1933) *Lancet*, ii, 575.

SANGHVI, L. D. AND KHANOLKAR, V. R.—(1949) *Ann. Eugen*, **15**, 52.

SANGHVI, L. D., RAO, K. C. M. AND KHANOLKAR, V. R.—(1955) *Br. med. J.*, i, 1111.

SATHE, V. R., KHANOLKAR, V. R., TALAGERI, V. R. AND PANSE, T. B.—(1957) *Indian J. med. Res.*, **45**, 401.

SNIJDERS, E. P. AND STRAUB, M.—(1923) Trans. Fifth Biennial Congress East. Ass. trop. Med., p. 779.

WELLS, C. R.—(1925) *Nav. med. Bull.*, **22**, 437.

WOELFEL, W. C., SPIES, J. W. AND CLIVE, J. K.—(1941) *Cancer Res.*, **1**, 748.

WYNDER, E. L. AND BROSS, I. J.—(1957) *Br. med. J.*, i, 1137.